# Traumatic Thumb Amputation: Case and Review

**Published:** 2015-03-24

**Authors:** Ryan Engdahl, Norman Morrison

**Affiliations:** ^a^Division of Plastic Surgery, New York Presbyterian Hospital, The University Hospital of Cornell and Columbia, New York; ^b^Division of Plastic Surgery, Harlem Hospital Center, Columbia University, New York

**Keywords:** thumb amputation, trauma, replantation, thumb reconstruction, toe to thumb

## DESCRIPTION

A 28-year-old healthy right-handed man arrived at the trauma bay after motorcycle crash with a crush-avulsion amputation of his left distal thumb. He was evaluated by trauma surgery and was stable.

## QUESTIONS

1. **What is the initial management of a traumatic digit amputation?**
2. **What factors should be taken into consideration for management of traumatic thumb amputation?**
3. **What are the general reconstructive options for a traumatic thumb amputation?**
4. **What are specific reconstructive options for a distal traumatic thumb amputation?**

## DISCUSSION

**Figure 1:**
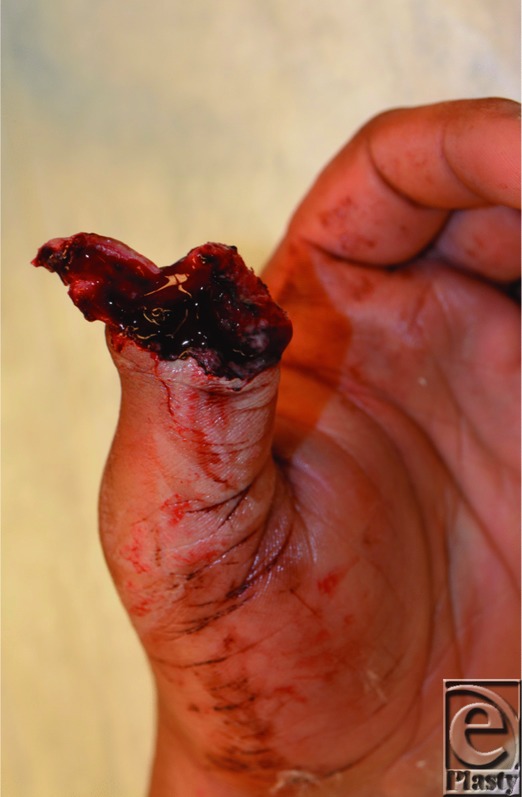
Distal thumb traumatic avulsion amputation.

**Figure 2:**
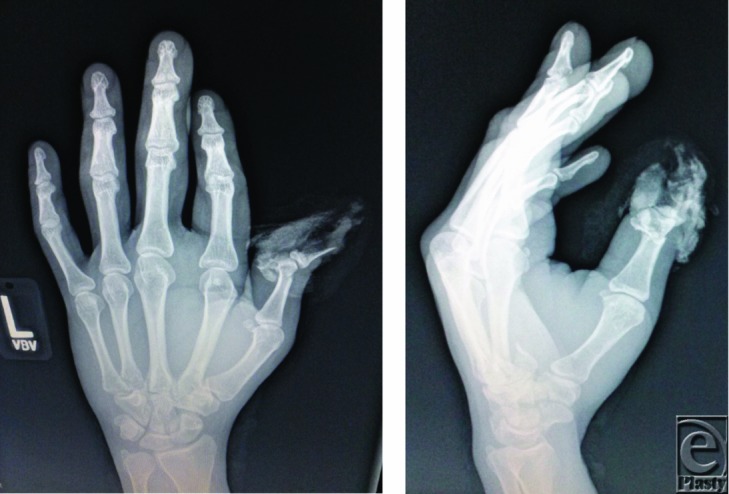
Radiographs of the distal thumb crush-avulsion revealing distal phalanx bone fragments and proximal phalanx transverse fracture.

**Figure 3:**
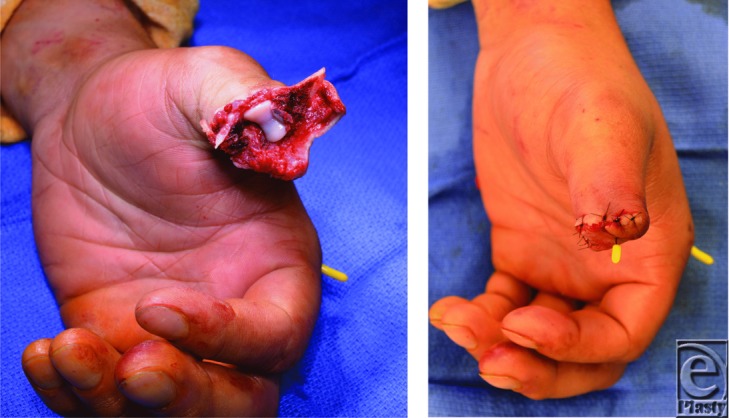
(*a*, *b*) Intraoperative debridement of the distal thumb avulsion injury. (*c*) Closure of the amputation and K-wire fixation of the thumb proximal phalanx transverse fracture and an index metacarpal fracture.

Initial management of a patient with traumatic digit amputation consists of exclusion of life-threatening injuries and patient stabilization. The amputated part should be wrapped in moist saline gauze and placed in a waterproof plastic bag in a container with ice and water. The part should not be placed directly on ice or directly immersed in saline, as this may injure and compromise the soft tissues. With any hand amputation, factors taken into consideration include age, occupation, hand dominance, time, and mechanism of injury. When considering replantation, transfer to a facility should be done as soon as possible and ischemia time of 12 hours of warm ischemia or 24 hours of cold ischemia is often tolerated. Replanting the thumb is indicated in clean-cut injury^1^; however, after crush injuries, the extent of tissue damage may be severe precluding replantation.^2^ Our patient exhibited a severe crush injury with loss of the majority of the amputated portion that precluded replantation (Figs 1–3).

**Figure 4:**
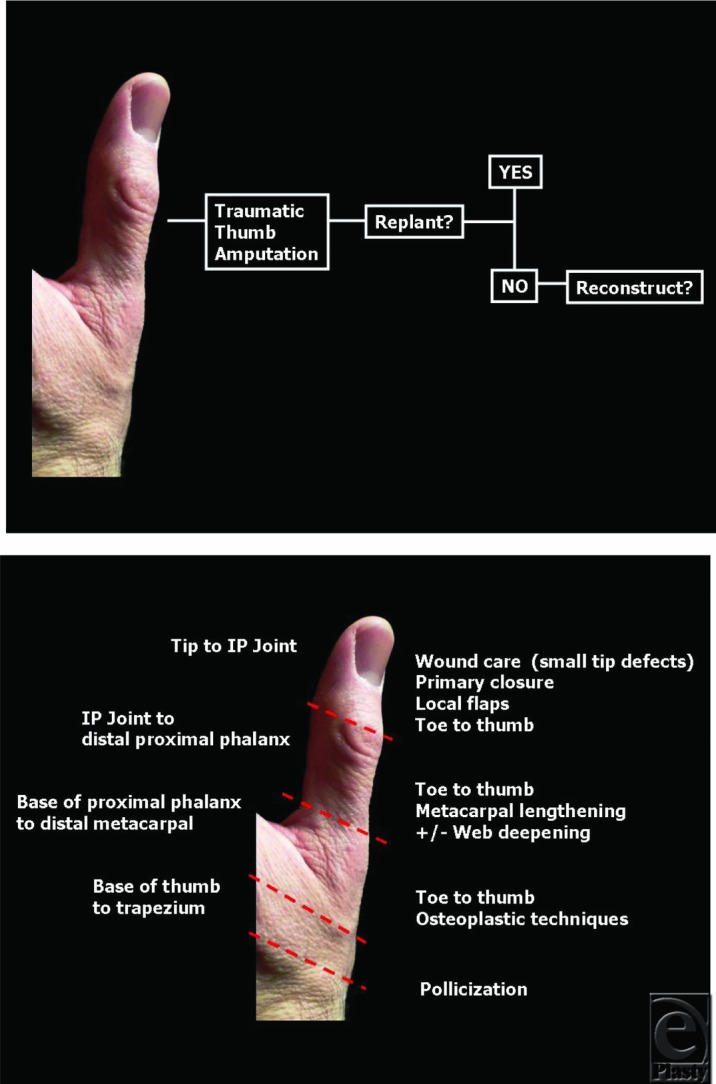
A simplified algorithm for a traumatic thumb amputation. Refer to the text for details. (*a*) Initial evaluation (*b*) Common reconstructive options by level of amputation.

Traumatic thumb amputation reconstructive goals include maintaining length and restoring function, sensation, and appearance. Algorithms can help guide thumb reconstruction options. However, given the complexities from patient factors to technical ones, management is often individualized.^3^ With initial traumatic thumb amputation, replantation is the first consideration.^4^^,^^5^ When replant is not possible, reconstructive options follow (Figs 4a and 4b), the timing of which can be short or delayed depending on the circumstance, patient factors, and surgeon preference.^6^^-^8

Reconstructive options can be divided into level of loss. In Figure 4b, levels of loss are divided into distal, middle, and proximal thirds of the thumb. Overall, amputations distal to the interphalangeal (IP) joint are often well tolerated. For smaller thumb tip or distal injuries, providing sensate and durable tissue may be all that is required. With very small tip defects, local wound care alone may allow them to heal.^1^ In those without exposed bone or structures, a skin graft might be an option as well. With larger tip defects or those with exposed structures or bone, a variety of flaps are options. Options include homodigital flaps such as V-Y advancements, or Moberg flaps, depending on the size and characteristics of the defect. Heterodigital flaps, such as cross-finger flaps, or neurovascular island flaps, such as the FDMA flaps, are used for larger defects. Whereas distal amputations (distal to the IP joint) may be well tolerated without significant hand function impairment, amputations proximal to the IP joint can result in significant functional loss.^1^ Beyond the thumb IP joint, the length of the remaining digit becomes a significant factor for thumb function. Classifications in this region include subtotal amputations (proximal to the IP joint) and total amputations (proximal to mid-portion of the proximal phalanx). Although many favor a toe-to-thumb transfer in these cases, methods that can be used to functionally lengthen the remaining digit include web space deepening (eg, Z-plasty). This may improve abduction and opposition. Functional techniques can be combined with distraction lengthening and flaps for coverage. Classic osteoplastic techniques include metacarpal lengthening, bone grafting, and flaps for soft-tissue coverage.

A toe-to-thumb transfer provides length and a digit. In general, toe-to-thumb transfers may play a role in reconstruction of amputations at or proximal to the mid-proximal phalanx, although they may be indicated for amputations from areas of the distal phalanx to the metacarpal shaft.^9^ For total or near total thumb amputations, choices include toe-to-hand transfer, osteoplastic reconstruction, or pollicization (Fig 4b). With total amputations (proximal to mid-portion of the proximal phalanx), the injury approaches the carpometacarpal joint. The status of this joint should be considered. Those with total amputations and preserved carpometacarpal joint, a toe-to-thumb transfer can be considered. Total metacarpal loss without a functional carpometacarpal joint is a difficult problem.^4^ Efforts often focus on making stable and sensate post that allows opposition. Techniques such as pollicization may play an important role.

The management of the loss of the distal digit (around the distal IP joint) varies from around the world.^1^ With clean-cut injury of the thumb at this level, replantation is usually the prime consideration. In those in whom replantation is not an option, such as our patient with a severe distal thumb crush-avulsion amputation, other reconstruction options may be considered. In this case, our patient after his acute injury underwent revision amputation with a good functional result and was not interested in further surgery (Figs 3a and 3b). More common in Asia, a toe-to-thumb reconstruction at this level, despite its technical complexity, may be an option for select patients. Although there is currently a lack of outcomes data comparing revision amputation with distal replantation or toe-to-thumb transfer at this level, success with distal reconstructions have recently renewed interest in this area.^7^^,^^10^^-^12
